# Reverse Electron Transfer by Respiratory Complex I Catalyzed in a Modular Proteoliposome System

**DOI:** 10.1021/jacs.2c00274

**Published:** 2022-04-05

**Authors:** John J. Wright, Olivier Biner, Injae Chung, Nils Burger, Hannah R. Bridges, Judy Hirst

**Affiliations:** Medical Research Council Mitochondrial Biology Unit, University of Cambridge, Cambridge CB2 0XY, U.K.

## Abstract

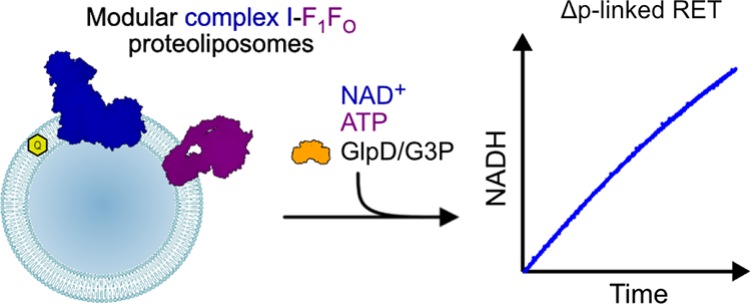

Respiratory complex I is an essential metabolic enzyme that uses the energy from NADH oxidation and ubiquinone reduction to translocate protons across an energy transducing membrane and generate the proton motive force for ATP synthesis. Under specific conditions, complex I can also catalyze the reverse reaction, Δp-linked oxidation of ubiquinol to reduce NAD^+^ (or O_2_), known as reverse electron transfer (RET). Oxidative damage by reactive oxygen species generated during RET underpins ischemia reperfusion injury, but as RET relies on several converging metabolic pathways, little is known about its mechanism or regulation. Here, we demonstrate Δp-linked RET through complex I in a synthetic proteoliposome system for the first time, enabling complete kinetic characterization of RET catalysis. We further establish the capability of our system by showing how RET in the mammalian enzyme is regulated by the active-deactive transition and by evaluating RET by complex I from several species in which direct assessment has not been otherwise possible. We thus provide new insights into the reversibility of complex I catalysis, an important but little understood mechanistic and physiological feature.

## Introduction

Respiratory complex I (NADH:ubiquinone oxidoreductase) is a redox-coupled proton pump central to oxidative phosphorylation in mitochondria and aerobic bacteria.^1,2^ By oxidizing NADH and reducing ubiquinone, it regenerates NAD^+^ to sustain crucial metabolic processes such as the tricarboxylic acid cycle and β-oxidation and provides electrons to the downstream complexes of the electron transport chain. The energy from the redox reaction is harnessed to translocate four protons across the inner mitochondrial membrane, or prokaryotic cytoplasmic membrane, contributing to the proton motive force (Δp) required for ATP synthesis and transport processes.^3^

As a major source of reactive oxygen species (ROS), complex I contributes significantly to cellular oxidative stress. In combination with its central metabolic role, this makes complex I dysfunctions one of the most frequent causes of mitochondrial disease.^4,5^ Furthermore, ROS production by complex I during “reverse electron transfer” (RET, Δp-driven ubiquinol:NAD^+^ oxidoreduction) has been shown to underlie the tissue damage occurring in strokes and heart attacks, during ischemia reperfusion (IR) injury.^6^ The physiological role of RET is now a major discussion in complex I function, with recent developments implicating the ROS produced by RET in redox signaling during inflammation,^7^ uncoupling of mitochondria in brown adipose tissue,^8^ oxygen sensing,^9^ and aging (in flies).^10^

Complex I catalysis has long been known to be reversible, but the RET redox reaction is energetically unfavorable, and so RET only occurs under specific metabolic conditions.^11^ There are two key prerequisites: a highly reduced Q-pool ([QH_2_] > [Q]) to provide the electrons and a high Δp to drive protons back through the complex in the opposite direction to forward catalysis. Together, the high QH_2_/Q ratio and high Δp provide the thermodynamic driving force required for RET: the proton transfer free energy (4Δp) overcomes the redox potential free energy (2Δ*E*) to drive electrons from QH_2_ (+80 mV) to NAD^+^ (−320 mV). When the two energies are equal, there is no net reaction, and catalysis switches from forward to reverse at this point.^1,12^

So far, studies of the kinetics and thermodynamics of RET have mainly focused on mammalian complex I, either in intact mitochondria or submitochondrial particles (SMPs).^12,13^ However, studies in these systems are all limited by complexity and inaccessibility: a multitude of proteins interact with the Q pool and Δp; mammalian mitochondria are difficult to manipulate genetically; and key kinetic parameters such as the membrane Q concentration cannot be varied. These limitations have precluded detailed mechanistic investigations of the RET reaction.

Here, we have engineered a modular, minimal system to study both “forward” and “reverse” catalysis by complex I under precisely defined conditions. We used established protocols to incorporate bovine complex I into proteoliposomes containing its highly hydrophobic natural substrate, ubiquinone Q_10_. Incorporation also of *Trypanosoma brucei brucei* alternative oxidase (AOX), a quinol-oxidizing enzyme, allowed us to study Q_10_-linked forward catalysis.^14,15^ Further incorporation of *Escherichia coli* ATP synthase (F_1_F_O_) then enabled us to use complex I to drive Δp-coupled ATP synthesis.^16^ Here, we replace AOX with the quinone-reducing enzyme glycerol 3-phosphate dehydrogenase (GlpD), which catalyzes glycerol-3-phosphate:ubiquinone oxidoreduction. By running the ATP synthase in reverse to generate Δp, we thus describe the first reconstituted system that efficiently catalyzes RET, enabling full kinetic characterization of the RET reaction and establishing a rationale for direction-dependent inhibition. We then demonstrate the potential of our system. First, we explore the regulation of RET to confirm that the deactive state, a resting state that forms in the absence of turnover, is neither RET competent nor can be reactivated by RET conditions. Second, we exploit the modular nature of our system to exchange complex I from *Bos taurus* for other homologues; we provide the first direct demonstration of RET (as NAD^+^ reduction) by complex I from *Mus musculus* and *Pichia pastoris* and thereby validate RET as a conserved mechanism across a range of species.

## Results

### GlpD-Catalyzed RET in SMPs

**Figure 1:**
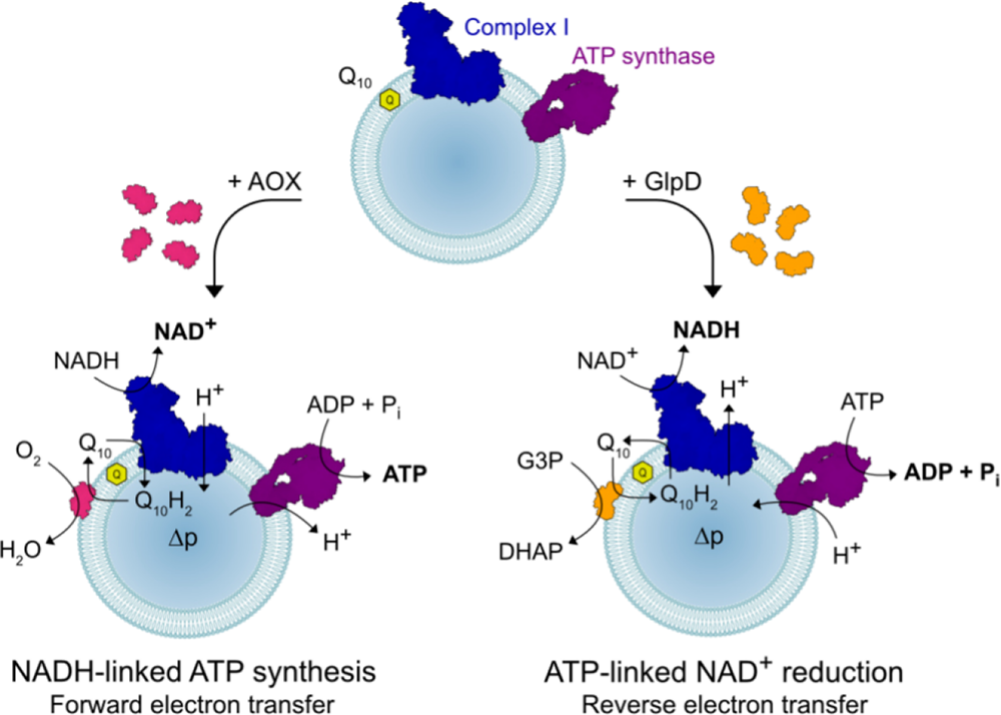
Schematic representation of the modular proteoliposomes system catalyzing forward or reverse electron transfer through complex I. Complex I and ATP synthase are co-reconstituted into liposomes with Q_10_. Then, AOX or GlpD is added to catalyze quinol oxidation or quinone reduction to drive forward or reverse electron transfer, respectively.

**Figure 2:**
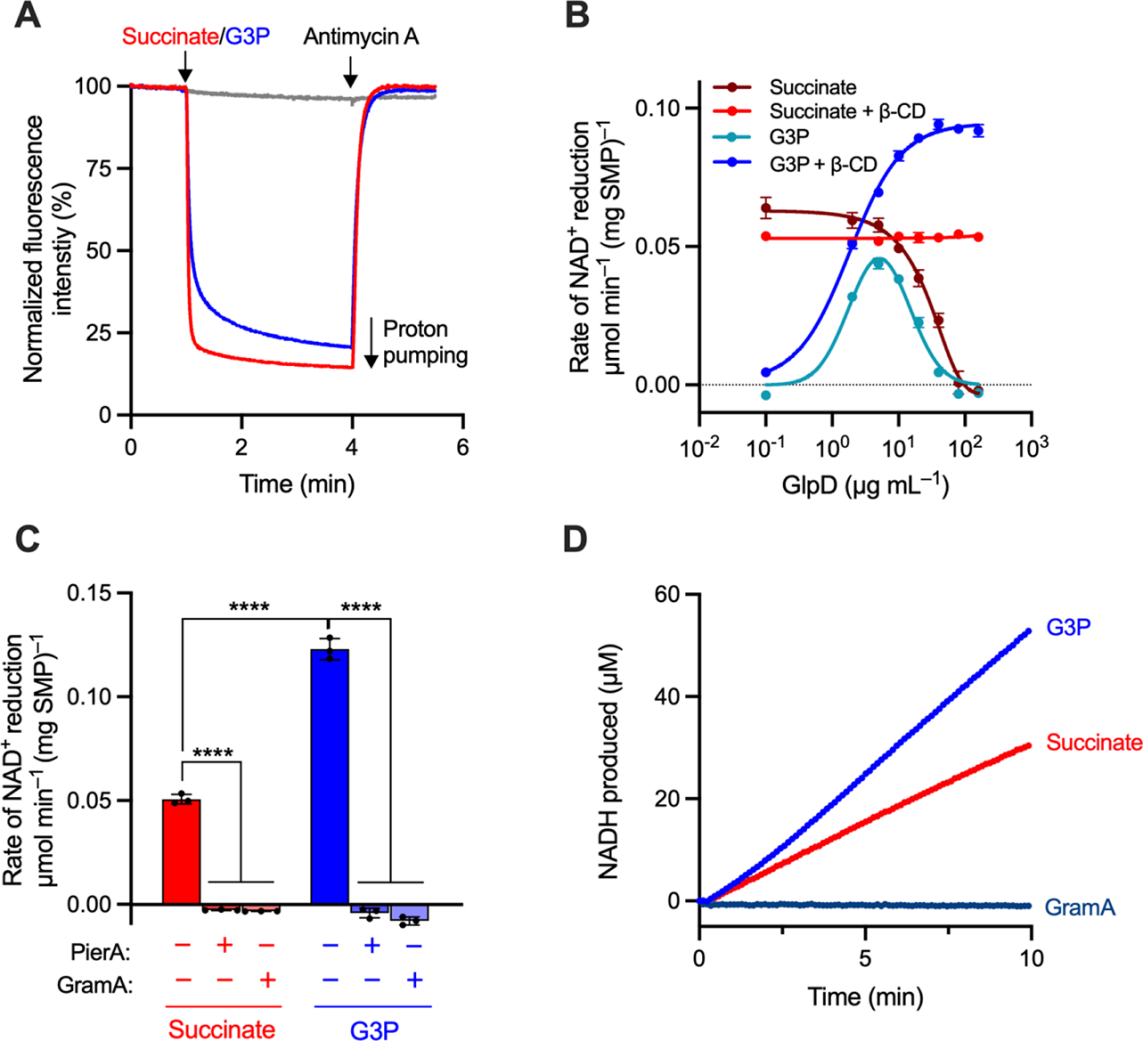
Optimization of GlpD-SMPs and demonstration of G3P-linked RET. (A) Proton pumping in GlpD-SMPs (catalyzed by CIII-CIV) monitored using 9-amino-6-chloro-2-methoxyacridine (ACMA) fluorescence. A total of 20 μg mL^–1^ SMPs were treated with 20 μg mL^–1^ GlpD, and catalysis was initiated by the addition of 1 mM succinate (red, CII-CIII-CIV) or 10 mM *rac*-G3P (blue, GlpD-CIII-CIV). A total of 2 μM antimycin A was added to inhibit CIII and stop proton pumping. Rates of GlpD-CIII-CIV catalysis are not optimized for the maximal rate. Addition of G3P does not support proton pumping in the absence of GlpD (gray). (B) Dependence of the rate of RET (monitored as NAD^+^ reduction) on GlpD concentration with and without β-CD (20 μg mL^–1^ SMPs). (C) RET in GlpD-SMPs is sensitive to piericidin A (PierA, 1 μM) and gramicidin A (GramA, 1 μg mL^–1^). Assays were performed as in (B), with 80 μg mL^–1^ GlpD. (D) Representative UV–vis assays displaying sustained RET. The data are mean averages of at least three technical replicates (± S.D.), with statistical significance from one-way ANOVA with Tukey’s multiple comparisons correction (****p < 0.0001).

We selected the monotopic glycerol 3-phosphate (G3P) dehydrogenase from *E. coli* (GlpD), which has previously been overexpressed, purified, and shown to bind liposomal membranes,^17,18^ to drive quinone reduction in our RET system (Figure 1, see Supplementary Text and Figure S1 for the characterization of GlpD). To first test the ability of GlpD to drive RET, we incorporated it into bovine SMPs, native-membrane vesicles well-established for studying both forward and reverse complex I catalysis.^12,19,20^ GlpD bound spontaneously to the SMP membrane (producing GlpD-SMPs) and, upon addition of G3P, catalyzed the reduction of Q_10_, activating downstream catalysis and proton pumping by complexes III and IV (Figure 2A). G3P- and succinate-linked catalysis by GlpD-SMPs were both sensitive to antimycin A, and no G3P-linked catalysis was observed without GlpD.

As the amount of GlpD added was increased, the rate of succinate/ATP-induced RET in GlpD-SMPs decreased, suggestive of membrane uncoupling (Figure 2B): the rate was constant up to ∼5 μg mL^–1^ GlpD but then declined rapidly. When RET was induced by G3P instead of succinate, the rate first increased with increasing GlpD but again declined rapidly above ∼5 μg mL^–1^. To avoid the decline, which we ascribed to the *n*-dodecyl-β-d-maltopyranoside (DDM, see Experimental Procedures) added with the GlpD, 2 mM heptakis(2,6-di-*O*-methyl)-β-cyclodextrin (β-cyclodextrin or β-CD)^21^ was added to sequester the DDM. Succinate-linked RET then became insensitive to GlpD concentration, and the rate of G3P-linked RET continued to rise to a plateau at ∼80 g mL^–1^ GlpD (Figure 2B). This first direct demonstration of G3P-linked RET through complex I was confirmed by inhibition of both reactions by piericidin A and gramicidin A (Figure 2C). Furthermore, the rate of G3P-linked RET exhibited by GlpD-SMPs (at high G3P concentrations) exceeds the rate of succinate-linked RET, indicating that succinate-linked RET in SMPs is limited by the rate of quinone reduction. Importantly, neither GlpD or its substrates or products exhibited any discernible inhibition of RET catalysis, with consistent linear traces observed once the maximal rate is established (Figure 2D).

### GlpD-Catalyzed RET in Proteoliposomes

**Figure 3:**
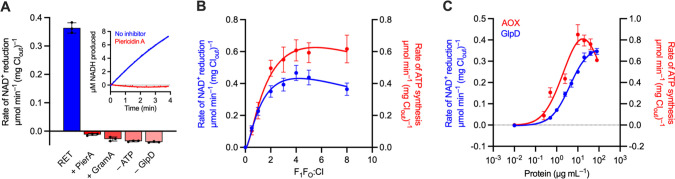
Demonstration and optimization of RET in complex I-containing proteoliposomes. (A) Rates of NAD^+^ reduction by proteoliposomes optimized for RET activity. Complex I was co-reconstituted with F_1_F_O_ ATP synthase at a 1:3 molar ratio, and then the proteoliposomes (5 μg mL^–1^ CI_out_) were incubated with 80 μg mL^–1^ GlpD and 2.5 mM β-CD in the assay solution. Complex I was first activated by the addition of 10 μM NADH prior to treatment with RET substrates. Piericidin A and gramicidin A were added at 1 μM and 1 μg mL^–1^, respectively. Inset shows a representative trace for NAD^+^ reduction by RET in the absence (blue) or presence (red) of piericidin A. (B) Dependence of the rates of NAD^+^ reduction and NADH-linked ATP synthesis on the number of moles of F_1_F_O_ ATP synthase added per mole of complex I during reconstitution; the complex I amount was constant. (C) Dependence of the rates of NADH-linked ATP synthesis (1 μg mL^–1^ CI_out_) and NAD^+^ reduction (5 μg mL^–1^ CI_out_) on increasing concentrations of AOX (molecular mass 33 kDa) and GlpD (56 kDa), respectively. 1 μg mL^–1^ AOX corresponds to 30 AOX per CI, and 1 μg mL^–1^ GlpD corresponds to 3.5 GlpD per CI. Assays were performed in the presence of 2.5 mM β-CD. See Experimental Procedures for standard assay conditions. Data are mean averages with error (± S.D.) values from triplicate technical replicates (including propagated error from all underlying measurements in (B)).

By combining co-reconstituted proteoliposomes containing complex I and ATP synthase with ATP to generate Δp by ATP hydrolysis and GlpD to reduce Q_10_ upon the addition of G3P, we demonstrate the first minimal system capable of catalyzing RET through complex I (Figure 3A, inset). Our approach avoids the challenging co-reconstitution of three proteins, as would be required for complex II (succinate:ubiquinone oxidoreductase) to drive succinate-linked RET. The G3P-linked RET observed is fully sensitive to both piericidin A and gramicidin A and was not observed when, for example, GlpD or ATP were omitted (Figure 3A). Furthermore, in our modular system, using the same CI-F_1_F_O_ proteoliposomes, addition of NADH, AOX, ADP, and phosphate drives NADH-linked ATP synthesis, whereas addition of NAD^+^, G3P, GlpD, and ATP drives RET. We refer to NADH:O_2_ oxidoreduction in complex I proteoliposomes as “forward electron transfer” (FET) and to the Δp-linked generation of ATP by the same reaction as NADH-linked ATP synthesis.

### Optimization of a Modular Proteoliposome System for Bidirectional Complex I Catalysis

The conditions of proteoliposome preparation and catalysis were optimized using complex I from *B. taurus* for NADH-linked ATP synthesis and RET:

(i) As for SMPs, GlpD spontaneously associated with the proteoliposomal membrane, and β-CD was needed to sequester its accompanying detergent. Maximal rates of RET were achieved at 1–2.5 mM β-CD (Figure S2A), below the excessive concentrations at which both RET (Figure S2A) and FET (Figure S2B) are affected. While β-CD did not benefit the maximal rate of NADH-linked ATP synthesis in AOX titrations (Figure S2C), it was beneficial at higher AOX concentrations. A total of 2.5 mM β-CD was used from hereon.
(ii) Titrating the amount of F_1_F_O_ ATP synthase in the reconstitution (at set complex I concentration) gave maximal rates for both NADH-linked ATP synthesis and RET (Figure 3B) at above three F_1_F_O_ ATP synthase per complex I. A standard ratio of three was therefore used from hereon.
(iii) The rates of NADH-linked ATP synthesis and RET were optimized by titrating the amounts of AOX and GlpD added, respectively, to the proteoliposomes in the presence of β-CD (Figure 3C). Both rates rise sharply at low concentrations. For AOX, NADH-linked ATP synthesis peaks at ∼10 μg mL^–1^ (10 μg AOX per μg of outward facing complex I (CI_out_)) then begins to decrease. A similar observation was previously ascribed to uncoupling by the DDM transferred along with the AOX.^16^ The inclusion of β-CD now argues against that interpretation, and a matching decrease was observed here for FET activity, both in the presence and absence of gramicidin A to intentionally abolish Δp (Figure S2D). A total of 10 μg mL^–1^ AOX was therefore used from hereon, to avoid the decrease, perhaps due to AOX aggregation. For GlpD, the rate of RET plateaus above ∼40 μg mL^–1^ (∼8 μg GlpD per μg of CI_out_). Similar mass ratios of AOX and GlpD are thus required, relative to complex I, to drive their reactions at the maximum rate. Concentrations of 80–100 μg mL^–1^ GlpD were used from hereon to ensure maximal quinone reduction during RET experiments.

### Determination of Kinetic Parameters for Quinol Oxidation during RET

**Figure 4:**
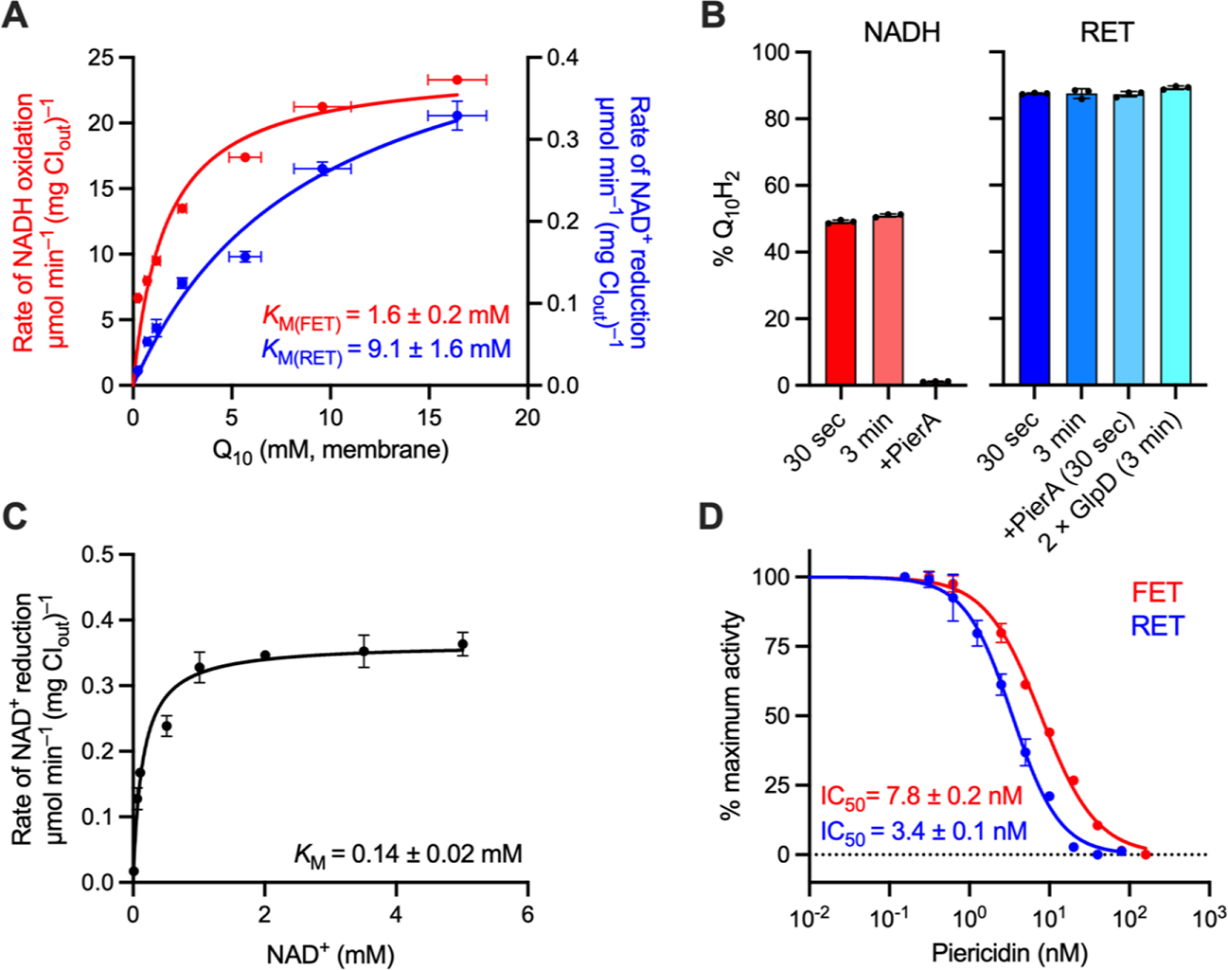
Kinetic parameters for FET and RET by bovine complex I in proteoliposomes. (A) Dependence of the rates of NADH oxidation (FET, blue) and NAD^+^ reduction (RET, red) on Q_10_ concentration in the membrane (1 mM Q_10_ (membrane) = 1 nmol Q_10_ per mg of phospholipid). RET activities were recorded with 100 μg mL^–1^ GlpD. The *V*_max_ values are 24.4 ± 0.85 and 0.50 ± 0.04 μmol min^–1^ (mg CI_out_)^−1^ (± S.E. of the fit). (B) Estimation of the level of Q-pool reduction for proteoliposomes reduced by NADH or catalyzing RET. For NADH measurements, no AOX was present and the Q-pool in 5 μg mL^–1^ CI_out_ proteoliposomes was reduced by addition of 200 μM NADH. For RET measurements, GlpD was present at 100 μg mL^–1^ and RET was initiated as described in Experimental Procedures before being quenched at the time specified. (C) Dependence of the rate of NAD^+^ reduction on NAD^+^ concentration. RET was measured under standard conditions with proteoliposomes reconstituted with 10 nmol Q_10_ (mg lipid)^−1^. The *V*_max_ value was 0.36 ± 0.01 μmol min^–1^ (mg CI_out_)^−1^. (D) Dependence of the rates of NADH oxidation (FET) and NAD^+^ reduction (RET) on piericidin A concentration. All measurements performed with 2 μg mL^–1^ CI_out_. See Experimental Procedures for standard assay conditions. All data are mean averages with error (± S.D.) values from triplicate technical replicates (including propagated error from underlying measurements in (A)).

Before this study, SMPs and intact mitochondria were the only systems in which RET could be measured routinely, but they do not allow manipulation of the Q_10_ concentration. Previously, the Q_10_ concentration was varied in CI-AOX proteoliposomes, relying on AOX to keep the quinone pool oxidized, to determine the Michaelis–Menten parameters for FET.^14,15^ Here, we extend the approach by using our modular system to determine the kinetics for FET and RET together using common proteoliposome preparations. Figure 4A compares the Michaelis–Menten curves for NADH oxidation (FET, CI:AOX, NADH, and O_2_) and NAD^+^ reduction (RET, GlpD:CI, G3P, NAD^+^, and ATP). The concentrations of complex I, phospholipids, and Q_10_ were individually determined for each preparation, to define its individual membrane-Q_10_ concentration. It was assumed that 1 mg of phospholipid occupies ∼1 μL, so that 1 nmol of Q_10_ per mg phospholipid is equivalent to 1 mM Q_10_ in the membrane.^22^ The *K*_M_ (Q_10_) of 1.65 ± 0.20 mM thereby determined for quinone reduction during NADH oxidation is consistent with values of 0.45–3.9 mM reported for complex I from *B. taurus*(14,15,23) and *Paracoccus denitrificans*,^24^ as well as with the value (2.45 mM) determined for NADH-linked ATP synthesis by the bovine enzyme.^16^ There was no substantial change in *K*_M_ (Q_10_) when gramicidin A was included to collapse Δp (1.65 ± 0.20 mM with gramicidin, 1.11 ± 0.15 mM without), but *V*_max_ was higher when it was present (24.4 ± 0.85 vs 18.3 ± 0.65 μmol min^–1^ (mg CI_out_)^−1^). For RET, *K*_M_ (Q_10_H_2_) for quinol oxidation by complex I was determined to be 9.13 ± 1.62 mM, a striking 5.5 times higher than *K*_M_ (Q_10_), with a *V*_max_ of 0.50 ± 0.04 μmol min^–1^ (mg CI_out_)^−1^.

Importantly, determination of *K*_M_ (Q_10_H_2_) for RET relies on GlpD holding the Q-pool reduced, an assumption supported by our observation that increasing the GlpD concentration affects neither the *K*_M_ (Q_10_H_2_) nor *V*_max_ (Figure S3A), so GlpD catalysis is not rate limiting. However, the rate of RET is also sensitive to the reduction potential of the Q-pool,^13^ a thermodynamic not kinetic effect (although the two are interdependent in our system). Therefore, the Q_10_H_2_/Q_10_ ratio was quantified using mass spectrometry.^25^ Following complex I preactivation (10 μM NADH, 3 min) and a 30 s or 3 min period of RET (with G3P, ATP, and NAD^+^), ∼90% of the Q-pool was found to be reduced (Figure 4B, RET) and this ratio did not change substantially when the GlpD concentration was increased or when piericidin A was added to inhibit complex I. The latter result suggests that Q_10_H_2_ oxidation by RET through complex I is not fast enough to alter the steady-state Q_10_H_2_/Q_10_ ratio substantially, so the incomplete reduction is due to a small proportion of the Q_10_ being inaccessible to GlpD (likely in multilamellar vesicles). In comparison, only ∼50% of the quinone was reduced when NADH was added (in the absence of AOX), and quinone reduction was abolished by piericidin A (Figure 4B, NADH). A substantial proportion of the quinone is thus probably in complex I-free liposomes (which are silent in RET assays). Together, the results confirm that the Q-pools in the RET-active population of proteoliposomes are near-fully reduced during RET catalysis.

We completed our basic kinetic characterization of RET as follows: (i) The dependence of the rate on NAD^+^ concentration (Figure 4C, *K*_M_ (NAD^+^) = 0.14 ± 0.02 mM) confirms that NAD^+^ concentration is not rate limiting in our standard conditions. (ii) The pH dependence of G3P-linked RET in proteoliposomes (Figure S3B) revealed a maximum at pH ∼8, and matching pH-dependencies were observed for both G3P- and succinate-linked RET in GlpD-SMPs. (iii) Using Q_10_-free liposomes, we demonstrated G3P-driven NAD^+^ reduction with either 100 μM decylubiquinone (DQ) or 100 μM Q_1_ but at lower rates (70%, DQ or 15%, Q_1_) than for ∼10 mM membrane-bound Q_10_ (Figure S3C).

Finally, we used our modular system to investigate the efficacy of a canonical complex I Q-site inhibitor in both directions of catalysis. The competitive binding mode of piericidin A has been established by combined structural, computational and kinetic approaches^23^ and studies using bovine heart SMPs^26^ have suggested it is a more potent inhibitor of RET than FET. Here, we directly assessed direction-dependent inhibition by piericidin A, with the Q-pool held either oxidized or reduced. Figure 4D confirms the IC_50_ value is lower during RET (IC_50_ = 3.4 ± 0.2 nM, Q-pool reduced) than during FET (IC_50_ = 7.8 ± 0.1 nM, Q-pool oxidized). However, the difference may result simply from competitive inhibition against a weaker binding substrate (the *K*_M_ for Q_10_H_2_ during RET is lower than for Q_10_ during FET): it does not necessarily mean that the inhibitor binding affinity is direction/state-dependent.

### RET Cannot Be Initiated from the Deactive State of Mammalian Complex I

**Figure 5:**
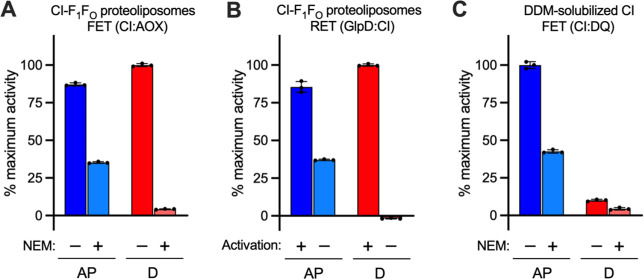
The active/deactive state of complex I assessed in co-reconstituted proteoliposomes. (A) The amounts of A and D complex I in a typical sample of as-prepared (AP) or deactivated (D) proteoliposomes were determined by sensitivity to derivatization with NEM (FET) or (B) by RET activity. (C) The proportion of A and D complex I determined in the DDM-solubilized enzyme. All measurements are normalized to the maximum rate for each set (CI:AOX = 35.5 μmol min^–1^ (mg CI_out_)^−1^, GlpD:CI = 0.618 μmol min^–1^ (mg CI_out_)^−1^, and CI:DQ = 14.3 μmol min^–1^ (mg CI)^−1^). All data are mean averages with error (± S.D.) values from three technical replicates.

Our proteoliposome system offers an unprecedented opportunity to confirm earlier proposals that the driving forces and conditions for RET are not able to reactivate “deactive” complex I.^19,27^ The active/deactive (A/D) transition of complex I is a biochemically^19,28−30^ and structurally^27,31^ defined phenomenon where a catalytically “ready-to-go” resting state, the A state, relaxes spontaneously but slowly into a pronounced resting D state in the absence of turnover; D requires reactivating by both NADH and ubiquinone to return to catalysis.^19^ Typically, investigations of the A/D transition are conducted in native preparations, owing to the much better stability of the membrane-bound enzyme compared to in detergent; this is particularly relevant when the enzyme is deactivated in a substrate-free incubation at 30 or 37 °C.^31^ Proteoliposomes now offer a stable environment in which A/D transitions can be studied in a minimal, well-defined system. Figure 5A shows results from the *N*-ethylmaleimide (NEM) assay used routinely to evaluate the relative amounts of A and D present.^29^ NEM derivatizes Cys39 of ND3 in the D state, rendering the enzyme inactive and resistant to reactivation.^29^ Here, the NEM assay showed that in a sample of “as-prepared” proteoliposomes, ∼36% of the FET activity was retained after NEM treatment and so ∼64% of the complex I was in D. This value is consistent with data from cryoEM analyses of bovine complex I,^32^ and it was consistent across several batches of proteoliposomes, both with and without ATP synthase, and unaffected by gramicidin A (Figure S4). Incubation of the proteoliposomes at 37 °C for 15 min increased the proportion of D to 97%. Importantly, the D enzyme regained full activity upon addition of NADH, confirming that complex I in proteoliposomes survives deactivation without loss of activity or integrity. In contrast, detergent-solubilized bovine complex I displayed a similar A/D ratio but underwent substantial unrecoverable loss of activity when incubated similarly at 37 °C to deactivate it (Figure 5C).

Figure 5B shows the rate of RET, as a proportion of the maximal rate possible, in as-prepared and deactivated proteoliposomes, measured with or without the addition of NADH to activate complex I before initiating RET. D-complex I in proteoliposomes, without reactivation, shows a complete absence of RET activity, whereas a short pre-activation by NADH restored the RET activity completely. Comparison of the RET rates suggests that 65% (in the example shown) of complex I is unable to catalyze RET in the as-prepared proteoliposomes, which matches the proportion of D enzyme determined by the NEM assay for the same sample (Figure 5A). Therefore, RET cannot be initiated from the D state, consistent with assignment of D to an off-cycle resting state,^27,31^ rather than an on-cycle intermediate.^33^ This result shows how the A/D transition regulates complex I activity and further demonstrates the capability of our modular system for investigations of the A/D transition in both directions of complex I catalysis.

### Testing the Ability of Complex I from Different Eukaryotes to Catalyze RET

**Figure 6:**
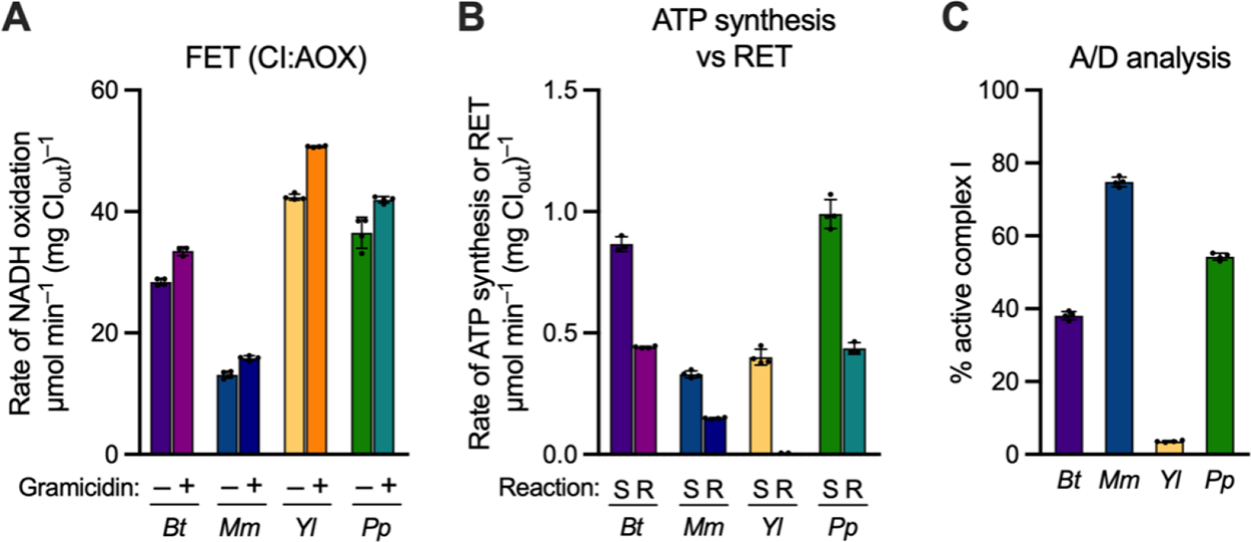
Comparison of catalysis by different species of complex I reconstituted into proteoliposomes. (A) Representative FET activities for proteoliposomes containing complex I from *B. taurus* (*Bt*), *M. musculus* (*Mm*), *Y. lipolytica* (*Yl*), and *P. pastoris* (*Pp*) in the presence and absence of 0.5 μg mL^–1^ gramicidin. (B) Comparison between rates of NADH-linked ATP synthesis (S) and RET (R) in proteoliposomes. Rates are calculated per mg of outward-facing complex I. (C) Analysis of the proportion of active complex I in as-prepared proteoliposomes, determined by sensitivity of catalysis to NEM, relative to a DMSO vehicle control. All data are mean averages with error (± S.D.) values from quadruplicate technical replicates.

It is not currently known whether RET is a general feature of all species of complex I or not. Until now, it has only been demonstrated in whole mitochondria or SMPs from mammals and *Drosophila melanogaster*(10,12,19,27) and in sub-bacterial particles (the bacterial equivalent of SMPs) from *P. denitrificans*,^34^ a close relative of the mitochondrial progenitor.^24^ Our modular system now provides the unique opportunity to test complexes I from different species, so the complexes from mouse heart (*Mus musculus*) and two yeast species (*Yarrowia lipolytica* and *Pichia pastoris*) were incorporated into proteoliposomes, in place of the bovine enzyme (Figure 6).

(i) Complex I from mouse heart^23,27,35^ shows close structural similarity to the bovine enzyme, and proteoliposomes created using mouse complex I and our standard protocol showed matching physical properties to their bovine counterparts (Table S1). Their FET activity was lower (Figure 6A), reflecting the lower activity of the detergent-solubilized mouse enzyme (∼10–12 μmol min^–1^ mg^–1^ for NADH:DQ oxidoreduction^23^ vs ∼20–25 μmol min^–1^ mg^–1^ for bovine complex I^31^), and their rates of ATP synthesis were thus also lower (Figure 6B). We now show directly, by Δp-linked NAD^+^ reduction (Figure 6B), that mouse complex I is capable of RET, with a rate consistent with these activities. Independently of complex I, the rates of ATP hydrolysis (Figure S5A) and Δp formation (Figure S5B) by the mouse proteoliposomes were also lower, suggesting lower retention of ATP synthase; further optimization was not pursued due to limitations in the availability of mouse heart tissue. Approximately 75% of the as-isolated mouse complex I was found to be in the A state (using the NEM assay), substantially higher than for bovine complex I, consistent with observations from cryoEM studies (Figure 6C).^35^
(ii) Complex I from *Y. lipolytica* and *P. pastoris* are similar in size and subunit composition to bovine complex I^36−38^ and have been incorporated into coupled proteoliposomes previously.^37,39^ Initial (standard) preparations of *P. pastoris* complex I proteoliposomes exhibited comparable rates of NADH oxidation and ATP synthesis (Figures 6A, B) to bovine proteoliposomes, plus consistent levels of ATP hydrolysis, ACMA fluorescence quenching, sensitivity to NEM (Figure 6C and Figure S5), and matching physical properties (Table S1). Near identical rates were also observed for RET in *P. pastoris* complex I (Figure 6B). This is the first direct demonstration of NAD^+^ reduction by RET for a yeast complex I. An initial heat treatment (37 °C, 15 min) was found to provide maximal rates of RET and ATP synthesis for *P. pastoris* complex I. The same treatment also stimulated bovine complex I, but it was less effective (∼15%) than for *P. pastoris*, where both rates increased by ∼50%. The close similarity to bovine complex I was further confirmed by the Michaelis–Menten parameters (Table S1, Figure S6A) for both FET and RET. The *K*_M_ values for both Q_10_ and Q_10_H_2_ were both lower for *P. pastoris*, but the *K*_M_ for Q_10_H_2_ was again significantly higher than for Q_10_ (ratio of 3.5 for *P. pastoris*).
(iii) Complex I from *Y. lipolytica* consistently showed remarkably high rates of NADH oxidation in proteoliposomes (Figure 6A and Table S1), considerably higher than for any other complex I tested. However, we have been unable to detect RET by *Y. lipolytica* complex I (Figure 6B and Figure S6B). Although rates of NADH-linked ATP synthesis from *Y. lipolytica* complex I are comparatively low, they are greater than from mouse complex I (which exhibits substantial RET), and the results for both ATP hydrolysis and Δp formation were consistent (Figure S5). Therefore, we considered whether the A/D ratio of complex I may preclude RET in *Y. lipolytica* complex I. Complex I from *Y. lipolytica* is known to have a lower energy barrier to deactivation than bovine complex I,^28,40^ and its deactive state is less developed structurally.^41,42^ According to the NEM assay, *Y. lipolytica* complex I was present entirely in D (Figure 6C), so it is possible that *Y. lipolytica* complex I cannot perform RET under the conditions tested here due to the ease with which it deactivates. Attempts to activate the complex with NADH before measuring RET were not successful, suggesting that the *Y. lipolytica* enzyme is unable to persist in an A-like state: a similar explanation has been proposed to explain why the P25L-ND6 variant of mouse complex I is also unable to catalyze RET.^27^

## Discussion

Complex I proteoliposomes combine the advantages of a purified enzyme system (simple and precisely defined composition and physical properties) with catalysis in a native-like membrane that sustains a proton-motive force (Δp). As such, they have already been used to investigate multiple aspects of complex I function including the kinetics of turnover and ubiquinone reduction,^14,15,24^ inhibition,^23,43,44^ ROS production,^45^ proton pumping,^39,46−48^ and ion transfer.^49^ The modular proteoliposome systems described here contain minimal respiratory chains designed to study complex I catalysis with the native, hydrophobic Q_10_ substrate incorporated in the membrane. They enable the study of both forward and (for the first time in proteoliposomes) reverse catalysis by complex I and of the kinetics, mechanism, and regulation of the RET reaction.

To drive RET in proteoliposomes, we incorporated GlpD to reduce the Q_10_ to Q_10_H_2_, to rapidly recycle the Q_10_ formed by complex I. We demonstrated that the Q-pool was held highly reduced during RET, focusing our kinetic data on the function of complex I, not GlpD. In contrast, succinate:ubiquinone oxidoreduction by complex II during RET in intact mitochondria could only hold the Q-pool ∼60% reduced.^13,25,27^ By accurately quantifying the concentrations of complex I, phospholipids and Q_10_, we determined the kinetic parameters for ubiquinol oxidation during RET, and by exploiting our modular system to switch GlpD for AOX, we simultaneously determined corresponding parameters for FET. Our observation that the *K*_M_ value for Q_10_ during FET is ∼5 times higher than for Q_10_H_2_ during RET indicates a clear kinetic bias for forward catalysis. Although this appears consistent with the substantially lower rates of catalysis observed for RET than FET, the rates have not been normalized for their thermodynamic driving forces or for the relative amounts of FET/RET-active complex I, precluding a fair comparison of turnover numbers. [We note that, for RET, complex I must be present alongside F_1_F_O_ in proteoliposomes capable of sustaining sufficiently high Δp, which is unlikely the case for all proteoliposomes capable of catalyzing FET.] The difference in *K*_M_ values provides a simple rationale for the relative efficacy of competitive complex I (Q-site) inhibitors, such as piericidin A, that inhibit RET more strongly than FET because they compete better against a less effective substrate. Notably, such inhibitors are emerging as a promising route to protect against the deleterious production of RET-ROS, for example, in IR injury.^50,51^ Our modular proteoliposome system now enables quantitative assessment of candidate drugs and inhibitors for both FET and RET, as well as mechanistic investigation of direction-dependent inhibitors. However, we note that our system is currently unsuited to study RET-driven ROS production, which is implicated in redox signaling and oxygen sensing,^7,9^ cardiac IR injury,^6^ and aging in flies.^10^ GlpD (and its mitochondrial homologue) also produces ROS,^52^ and its high concentration in our assays causes substantial background ROS production.

Due to its energetic demands, the rate of RET is exquisitely dependent on both the Q-pool potential and Δp, as demonstrated by measurements of the thermodynamic driving force for RET-associated ROS production in whole mitochondria.^13^ In our modular system, Δp can be created by ATP hydrolysis, and the directionality of complex I catalysis is determined by the choice of partner enzyme (AOX or GlpD) that, through essentially irreversible catalysis, sets the Q-pool (near) fully oxidized or reduced. As a result, our “binary” system can only be switched from one direction to the other, not titrated between the two extremes. The effects of the Q-pool potential and the Δp can therefore be probed independently of one another.

A lack of diversity in experimental systems able to support RET means it has so far only been studied widely in mammalian complex I. Direct observations of RET have typically been investigated using SMPs, based on the accessibility of the outward-facing substrate binding sites, their ability to sustain high Δp, and ease of monitoring NAD^+^ reduction.^12,19^ Although SMPs from yeast species have been described,^53^ they have not been used to demonstrate RET, and measurements on *D. melanogaster* have focused only on RET-ROS production. The only non-mammalian species for which RET has been observed directly is the bacterium *P. denitrificans*.^24,34^ Here, we provide the first direct evidence of RET by complex I from two new species, *M. musculus* (mouse) and the yeast *P. pastoris*. Although our results further support the conservation of reversibility in complex I catalysis, complex I from *Y. lipolytica* was not able to catalyze RET under the conditions tested, highlighting differences in complex I regulation between species. Previously, we observed that the P25L-ND6 complex I mouse variant is protected against IR injury because P25L-ND6 complex I, despite being capable of catalyzing FET normally, is unable to catalyze RET-ROS formation.^27^ The protective effect was assigned to the increased tendency of the enzyme to drop into D-like states, as a result of the mutation perturbing the stability around the π-bulge in ND6, a hallmark of D. Notably, this protective mechanism relies on the conditions for RET not being capable of reactivating the D enzyme to initiate RET, which we have demonstrated here to be the case. Parallels between P25L-ND6 mouse and *Y. lipolytica* complex I thus lead us to ask if RET is prevented by the same mechanism.

It has long been known that the A state in *Y. lipolytica* complex I is less stable than its mammalian counterpart,^40^ but the reasons are not well understood. Structural data revealed that, although the extent of the deactive transition is less in *Y. lipolytica*, important hallmarks of the deactive enzyme are present in the as-prepared enzyme,^41^ including the characteristic π-bulge in TMH3 of the ND6 subunit, and this was also conserved in structural analyses of a proposed “turnover” state.^42^ Therefore, rapid deactivation may explain the inability of *Y. lipolytica* complex I to catalyze RET. We note that, in mammalian complex I, conformational rearrangements reposition the NDUFA10 and NDUFA5 supernumerary subunits relative to each other during the A/D transition,^35,54^ and the absence of NDUFA10 in *Y. lipolytica* may contribute to its low energy barrier for deactivation. However, *P. pastoris* lacks NDUFA10^37^ and yet displays a substantial proportion of complex I in the A state, and *P. denitrificans* lacks both NDUFA10 and NDUFA5 and appears fully in the A state.^24^ Therefore, there is a clear cross-species correlation between the ability to catalyze RET and the existence of the A state but no correlation with the presence of NDUFA10 and NDUFA5. Finally, mass spectrometry data have revealed that an arginine residue on a loop in subunit NDUFS7 (Arg77 in *B. taurus*), which forms part of the ubiquinone binding site and changes conformation between the A and D states, is post-translationally hydroxylated in *B. taurus*, *M. musculus*, *P. pastoris*, and *P. denitrificans*(55,56) but not in *Y. lipolytica*.^57^ This is a second clear cross-species correlation: between the ability to catalyze RET and Arg77 hydroxylation. In detail, however, there is no correlation between Arg77 hydroxylation and the conformation of the loop that carries it, as in structures of *Y. lipolytica* complex I, this specific loop is found in a conformation matching the mammalian A (not D) conformation. Extending the lists of species with RET-active (or inactive) complexes I is thus required to better understand these correlations and to elucidate the subtle structural variations that occur and that may underpin mechanisms of complex I catalysis and regulation.

## Conclusions

In summary, we have demonstrated Δp-linked reverse electron transfer by complex I in a proteoliposome system for the first time and exploited the well-defined and quantifiable nature of our system to determine previously inaccessible kinetic parameters. The modular nature of our proteoliposome system allows different forms of complex I to be incorporated and analyzed during FET, RET, and the A/D transition. Our system thereby enables investigations of complex I from less experimentally developed species, and opens the door to further detailed characterization of the effects of mechanistic and clinically relevant mutations in both directions of catalysis.

## Experimental Procedures

### GlpD Growth, Expression, and Purification

The *E. coli* gene encoding GlpD (*glpD*) was synthesized and inserted into a pET-28a expression vector (containing an N-terminal His_6_-tag) using the *Nde*I and *Bam*HI restriction sites by a commercial service (GenScript). Two stop codons (TAG and TGA) were added at the C-terminus. The GlpD-pET28a plasmid was transformed into *E. coli* strain BL21(DE3) pLysS, and then GlpD was overexpressed and purified using a method adapted from Yeh et al.^17^ Starter cultures were grown overnight at 37 °C in 200 mL LB broth supplemented with 50 μg mL^–1^ kanamycin and 25 μg mL^–1^ chloramphenicol. The cells were diluted 100-fold into terrific broth (containing 50 μg mL^–1^ kanamycin and 25 μg mL^–1^ chloramphenicol) and grown to OD_600_ ≈ 0.7 at 37 °C. GlpD overexpression was induced by the addition of 1 mM isopropyl β-d-1-thiogalactopyranoside (IPTG), and the culture was grown for a further 4 h. Cells were harvested at 6000 *g* for 10 min at 4 °C, washed in 50 mM HEPES (pH 7.4), 100 mM NaCl then pelleted and stored at −80 °C.

Cells from 12 L of culture were thawed and resuspended in 120 mL 50 mM HEPES (pH 7.4), 100 mM NaCl, and then 1 mM MgSO_4_, 0.5 mM PMSF, and a few crystals of DNase1 were added. The cells were lysed with a French press (two passes at 12,000–15,000 psi). Cell debris and unbroken cells were removed by centrifugation (8000 *g*, 20 min, 4 °C), and the supernatant was ultracentrifuged at 100,000 *g* (60 min, 4 °C). The membrane pellets were resuspended in 120 mL resuspension buffer and homogenized, and 40 mL aliquots were frozen in liquid N_2_. Individual aliquots were thawed, and the membranes were solubilized on ice for 60 min by the addition of 1.3% octyl glucoside. The suspension was clarified by ultracentrifugation (100,000 *g*, 60 min, 4 °C), filtered (0.22 μm), and loaded onto a 5 mL HisTrap HP column equilibrated with GlpD Buffer A (20 mM HEPES (pH 7.4), 20 mM KCl, 200 mM NaCl, 0.05% DDM). The column was washed with 37.5% Buffer B (Buffer A + 400 mM imidazole), and then GlpD was eluted with 100% Buffer B. GlpD-containing fractions were collected, pooled, concentrated in an Amicon Ultra-15 (10 MWCO), and dialyzed overnight at 4 °C against 2 L of Buffer A (3.5 kDa MWCO). 20% glycerol was added for storage at −80 °C.

### Purification of Complex I

Complex I was purified from *B. taurus*,^15^*M. musculus*,^35^ and *Y. lipolytica*(46) using established protocols with minor alterations. *P. pastoris* complex I was purified using a method adapted from published procedures.^37,58,59^ See Supporting Information for further details.

### Purification of AOX

Recombinantly expressed AOX was prepared from *E. coli* membranes by solubilization with octyl-glucoside and purified by Twin-Strep tag affinity chromatography as described previously.^15^

### Purification of ATP Synthase from *E. coli*

ATP synthase from *E. coli* strain DK8 and the pBWU13-βHis plasmid was prepared as described previously.^16,60^

### Preparation of Submitochondrial Particles

SMPs supplemented with cytochrome *c* were prepared as described previously^61,62^ and suspended in 10 mM MOPS (pH 7.5), 50 mM KCl, and 250 mM sucrose.

### Preparation of Proteoliposomes

Proteoliposomes were prepared using a protocol adapted from Biner et al.^16^ A total of 10 mg of lipids (8:1:1, 1,2-dioleoyl-*sn*-glycero-3-phospho-choline (DOPC), 1,2-dioleoyl-sn-glycero-3-phospho-ethanolamine (DOPE), and 18:1 cardiolipin (CDL)) in chloroform were combined with Q_10_ from a chloroform stock (10 nmol (mg lipid)^−1^), and the solvent was evaporated off with N_2_ before drying under vacuum for >1 h. The lipids were hydrated in 1 mL of proteoliposome buffer (10 mM MOPS (pH 7.5), 50 mM KCl) for 30 min with frequent vortexing and then sonicated on ice using a Q700 probe sonicator (QSonica) equipped with a 1.6 mm microtip (60% amplitude, 2.5 min (15 s on/30s off)). The resulting liposomes (10 mg mL^–1^) were then reconstituted (typically at 2.5 or 5.0 mg lipid) by partially solubilizing the lipids with 0.5% (final concentration) sodium cholate for 10 min on ice. Complex I (50:1 (w/w) lipid to protein) was then added, alongside F_1_F_O_ ATP synthase (molar ratio of 3 F_1_F_O_ to 1 CI, giving final of 20:1 (w/w) total lipid to protein ratio), and incubated for a further 10 min. The detergent was removed using a PD10 desalting column (Cytiva). The sample was centrifuged (150,000 *g*, 1 h, 4 °C) followed by resuspension in proteoliposome buffer. Proteoliposomes were kept on ice or at 4 °C before use; no loss in activity was observed during overnight storage.

### Characterization of Proteoliposomes

Total phospholipid contents were determined using the Ames phosphate assay.^14^ Total Q_10_ content was determined using an HPLC system equipped with a Thermo Scientific Dionex Ultimate 3000RS Electrochemical Detector.^16^ Q_10_ concentrations are expressed in mM relative to the volume of the membrane, where 1 mM Q_10_ (membrane) = 1 nmol Q_10_ per mg of phospholipid.^22^ The redox state of the Q pool was determined using a mass spectrometry assay following Q_10_ extraction.^25^ Complex I content and orientation in proteoliposomes were estimated by comparing the rate of NADH:APAD^+^ oxidoreduction to the rate from a standard detergent-solubilized complex I sample, assayed in proteoliposome buffer with 100 μM NADH, 500 μM APAD^+^, 500 nM piericidin A and 0.2% (w/v) DDM.^15,16,24^ Proteoliposome APAD^+^ reduction rates were measured without DDM under otherwise identical conditions, with 15 μg mL^–1^ alamethicin added to determine the (inside/outside) orientation.

### NADH Oxidation Assays

NADH:O_2_ oxidoreduction activity was measured spectrophotometrically (ε_340–380_ = 4.81 mM^–1^ cm^–1^) using a SpectraMax plus 348 96-well plate reader (Molecular Devices). Standard measurements were carried out in 10 mM MOPS (pH 7.5), 50 mM KCl at 32 °C, with complex I (0.5 μg mL^–1^, outward facing) and AOX (10 μg mL^–1^). Turnover was initiated by addition of 200 μM NADH. Uncoupled rates were determined with 0.5 μg mL^–1^ gramicidin A. Inhibition measurements were conducted with 2 μg mL^–1^ outward facing complex I and 2.5 mM β-CD for both FET and RET systems. We note that β-CD has the capacity to interact with hydrophobic inhibitors and artificially increase the absolute IC_50_ values; here, we observed the ratios of IC_50_ values in FET and RET to be unaffected.

### NAD^+^ Reduction Assays

For SMP experiments, (GlpD)-SMPs were used at 20 μg protein mL^–1^ in 10 mM MOPS (pH 7.5), 50 mM KCl. They were activated by 3 min incubation with 10 μM NADH at 32 °C prior to the addition of RET substrates (1 mM ATP, 1 mM MgSO_4_, 1 mM NAD^+^ plus 10 mM succinate or 120 mM *rac*-G3P), and the reaction followed spectrophotometrically at 340 and 380 nm. For standard proteoliposome experiments, CI-F_1_F_O_ proteoliposomes (5 μg mL^–1^ outward facing complex I) were treated with 2.5 mM β-CD and GlpD (80–100 μg mL^–1^), preactivated by 3 min incubation with 10 μM NADH at 32 °C, and then RET was initiated as in the SMP experiments. In experiments with *P. pastoris* complex I, liposomes were incubated at 37 °C for 15 min before activation with NADH.

### NEM Assays for Active/Deactive Complex I Determination

Deactivation of complex I was achieved by incubation of proteoliposomes (200 μg mL^–1^ outward facing complex I) or DDM solubilized complex I (17.1 mg mL^–1^) at 37 °C for 15 min. *N*-Ethylmaleimide (NEM) was dissolved at 400 mM in DMSO before dilution to 100 mM in proteoliposome buffer. Soluble complex I was diluted to 200 μg mL^–1^, and samples were treated with 1 mM NEM (or the equivalent volume of 25% DMSO) and incubated on ice for 20 min before determining the NADH:O_2_ or NADH:DQ activity.^31^ NADH:O_2_ activity was performed as described above. NADH:DQ activity was performed with 0.5 μg mL^–1^ complex I, 200 μM NADH, 200 μM DQ, and 0.15% (w/w) of each of asolectin (Soy bean, 20% phosphatidylcholine) and CHAPS in 20 mM Tris–HCl (pH 7.5 at 32 °C).

### Q_1_ Reduction Assays for GlpD Activity

GlpD (0.1 μg mL^–1^) was incubated in 10 mM MOPS (pH 7.5), 50 mM KCl at 32 °C with 200 μM Q_1_. Where indicated, 0.15% (w/w) of each of asolectin and CHAPS was added to the reaction mixture. Catalysis was initiated by the addition of 120 mM *rac*-G3P, and the reduction of Q_1_ was monitored at 275 nm (ε = 13.7 mM^–1^ cm^–1^)^63^ using a quartz plate.

### ATP Synthesis Assays

ATP synthesis was performed as previously described,^16^ with slight modifications. The assay comprised 20 mM Tris–PO_4_ (pH 7.4), 5 mM MgCl_2_, 2.5 mM β-CD, 50 μM ADP, 20 μL mL^–1^ luciferase reagent (ATP Bioluminescence Assay Kit CLS-II, Roche), 10 μg mL^–1^ AOX, and 1 μg mL^–1^ of complex I (outward-facing). ATP synthesis was stopped by the addition of 1 μg mL^–1^ gramicidin A.

### ATP Hydrolysis Assays

ATP hydrolysis was performed at 32 °C using a coupled assay system that oxidizes NADH (measured at 340 and 380 nm) in response to the production of ADP.^64^ Equivalent volumes of proteoliposomes were diluted into 10 mM MOPS (pH 7.5), 50 mM KCl containing 1 mM ATP, 1 mM MgSO_4_, 2 mM K_2_SO_4_, 200 μM phosphoenolpyruvate (PEP), 50 μg mL^–1^ lactate dehydrogenase (LDH) from bovine heart, 40 μg mL^–1^ pyruvate kinase (PK) from rabbit muscle, and 2 μM piericidin A to prevent complex I mediated NADH oxidation.

### ACMA Fluorescence Quenching Assays

ACMA assays to assess proton pumping qualitatively were performed using a RF-5301PC spectrofluorometer (Shimadzu) at 32 °C. Proteoliposomes were transferred to buffer (10 mM MOPS (pH 7.5), 50 mM KCl) containing 0.5 μM ACMA and 0.1 μM valinomycin with constant stirring. For NADH:O_2_ ACMA assays, AOX was added at 5 μg mL^–1^ and proton pumping initiated by addition of 500 μM NADH. Δp was dissipated by the addition of 10 μg mL^–1^ alamethicin. For ATP hydrolysis, 1 mM MgSO_4_ was added to the buffer and proton pumping was initiated by the addition of 1 mM ATP.

## Supporting Information Available

Supplementary experimental procedures (purification of complex I from different species) and results (characterization of the quinone reductase GlpD); additional details on proteoliposome reconstitutions and kinetic fitting; and supplementary figures showing the characterization of GlpD, the effects of β-CD, quinone and pH, measurements with uncouplers, comparisons of ATP hydrolysis, and additional Q_10_ titrations (PDF)

## Abbreviations

β-CD: β-cyclodextrin
ACMA: 9-amino-6-chloro-2-methoxyacridine
AOX: alternative oxidase
C(I–IV): complex (I–IV)
DDM: *n*-dodecyl-β-d-maltopyranoside
DHAP: dihydroxyacetone phosphate
DQ: decylubiquinone
FET: forward electron transfer
G3P: glycerol 3-phosphate
GlpD: glycerol 3-phosphate dehydrogenase
NEM: *N*-ethylmaleimide
P_i_: inorganic phosphate
RET: reverse electron transfer
ROS: reactive oxygen species
SMP: submitochondrial particles.
