# Supplementary material for: Reverse Electron Transfer by Respiratory Complex I Catalyzed in a Modular Proteoliposome System
Source: J Am Chem Soc. 2022 Apr 5;144(15):6791–801. doi: 10.1021/jacs.2c00274 (PMC9026280; doi:10.1021/jacs.2c00274)
Supplement: Supplementary file 1 — ja2c00274_si_001.pdf [file ja2c00274_si_001.pdf]

Supplementary Information

Reverse electron transfer by respiratory complex I catalyzed in a modular proteoliposome system

John J. Wright, Olivier Biner, Injae Chung, Nils Burger, Hannah R. Bridges & Judy Hirst*.

Medical Research Council Mitochondrial Biology Unit, University of Cambridge, Cambridge CB2 0XY, UK
*Corresponding Author: Judy Hirst (jh@mrc-mbu.cam.ac.uk)

Supplementary Text

Purification of complex I from different species:

Complex I from *Bos taurus*: Mitochondrial membranes were prepared from bovine heart tissue as described previously,[1] followed by complex I solubilization and purification according to our established protocol,[2] with the minor alteration of the gel filtration buffer containing 150 mM NaCl rather than 200 mM.

Complex I from *Yarrowia lipolytica*. Membranes from *Y. lipolytica* strain GB10 were prepared as described previously.[3,4] Purification of complex I from *Y. lipolytica* membranes was achieved using the protocol described by Brandt and co-workers[5] with minor modifications: solubilized membranes (~800 mg) were loaded onto two 5 mL HisTrap HP columns (Cytiva) in series and the eluted complex I fractions were concentrated and loaded onto a Superose 6 Increase column (Cytiva) for size exclusion chromatography. Glycerol was added to the pure complex I sample at a final concentration of 20% (v/v).

Complex I from *Pichia pastoris*. *P. pastoris* complex I was purified using a method adapted from previously published protocols for *P. pastoris*[6] and *Y. lipolytica*[3,4]. *P. pastoris* strain X33 was grown as described.[6] For the membrane preparation, cells were diluted to 500 g L^{-1} in 20 mM MOPS (pH 7.2), 400 mM sorbitol, 5 mM EDTA, 5 mM benzamidine hydrochloride, 5 mM ε-aminocaproic acid, 2% bovine serum albumin (BSA). 800 μM phenylmethylsulfonyl fluoride (PMSF) was added to the sample before disruption using a Dyno®-Mill bead mill (2 passes at ~10 mL min^{-1}). Unbroken cells and debris were removed by centrifugation (6,000 g, 15 minutes). The supernatant was collected and ultracentrifuged (208,000 g, 60 minutes) and the membrane pellet was retained. The membranes were resuspended and homogenized in ~800 mL of 20 mM MOPS (pH 7.2), 400 mM sorbitol, 5 mM ε-aminocaproic acid, 5 mM benzamidine, before a second ultracentrifugation step (208,000 g, 45 minutes). The pellets were again resuspended and homogenized in ~400 mL of 20 mM MOPS, 400 mM sorbitol, 5 mM ε-aminocaproic acid, 5 mM benzamidine (pH 7.2). A final ultracentrifuge step (208,000 g, 35 minutes) was performed before rehomogenization in 20 mM NaH_2PO_4 (pH 7.45), 50 mM NaCl. Washed membranes were aliquoted and frozen in liquid N_2.

Defrosted membranes were diluted to 20 mg protein mL^{-1} with 20 mM NaH_2PO_4 (pH 7.4), 50 mM NaCl. Two EDTA-free protease inhibitor cocktail tablets (Roche) were added per 50 mL of membrane suspension and the membranes were solubilized on ice for 30 minutes following the addition of 2.3% DDM. The sample was centrifuged (208,000 g, 45 minutes) and the supernatant was treated with 400 mM NaCl (s) and 20 mM imidazole from a neutralized 1 M stock. The sample was filtered (0.22 μm) and loaded onto a 40 mL Ni-sepharose FF column pre-equilibrated with 20 mM NaH_2PO_4 (pH 7.2), 400 mM NaCl, 42 mM imidazole, 0.02% asolectin, 0.02% 3-((3-cholamidopropyl) dimethylammonio)-1-propanesulfonate (CHAPS), 0.1% DDM. The column was washed with ~100 mL of the same buffer, before elution with 20 mM NaH_2PO_4 (pH 7.2), 400 mM NaCl, 140 mM imidazole, 0.02% asolectin, 0.02% CHAPS, 0.1% DDM. Complex I from *P. pastoris* was found to have a natural affinity for Ni-sepharose and required no artificial His-tag for its purification. Fractions containing complex I were concentrated with an Amicon Ultra-15 (100 MWCO) spin concentrator and injected onto a Superose 6 Increase 10/300 gel filtration column, pre-equilibrated with 20 mM MOPS (pH 7.45), 150 mM NaCl, 0.05% (w/v) DDM, 10% (v/v) glycerol. Fractions containing pure complex I were concentrated as before. 20% glycerol was added to the sample, which was then aliquoted and frozen in liquid N_2.

Complex I from *Mus musculus*. C57BL/6 mice were sacrificed by cervical dislocation in accord with the UK Animals (Scientific Procedures) Act 1986 (PPL: P6C97520A, approved by the local ethics committee and the UK Home Office). Hearts were excised, then immersed immediately in ice-cold buffer containing 10 mM Tris-HCl (pH 7.4 at 4 °C), 75 mM sucrose, 225 mM sorbitol, 1 mM EGTA, and 0.1% (w/v) fatty acid-free BSA, supplemented with one cOmplete™ EDTA-free protease inhibitor tablet (Roche) per 50 mL buffer. All subsequent steps were performed at 4 °C. Mitochondrial membranes were prepared from heart tissues,[7] then complex I was prepared as described previously,[7] with minor modifications. Briefly, membranes were diluted to 5 mg protein mL^{-1} in 20 mM Tris-HCl (pH 7.4 at 4 °C), 1 mM EDTA, and 10% glycerol, supplemented with one cOmplete™ EDTA-free protease inhibitor tablet per 50 mL buffer. Proteins were solubilized from the membranes by addition of 1% DDM, stirred for 30 minutes on ice, and centrifuged. Then, the detergent-solubilized complexes were separated by ion-exchange chromatography using a 1 mL Hi-Trap Q Hp column (Cytiva). The complex I–containing fractions were pooled and concentrated to 100 μL using an Amicon Ultra concentrator (100 MWCO.), and the sample applied to a Superose 6 increase 5/150 column (Cytiva) and eluted in 20 mM Tris-HCl (pH 7.14 at 20 °C), 150 mM NaCl, 10% ethylene glycol, and 0.05% DDM. The complex I fractions were pooled and concentrated as above, then treated with 20% glycerol before flash freezing in liquid N_2 and storage at –80 °C.

Results: Characterization of the quinone reductase GlpD for RET catalysis.

In order for GlpD to be considered as a candidate for driving quinone reduction for RET experiments, it must fulfil two main requirements. First, it must be capable of sufficient turnover that oxidation of the quinol by complex I is rate limiting. Second, it must not be sensitive to standard inhibitors and uncouplers used as controls in complex I studies. We therefore tested the suitability of GlpD to meet these requirements. GlpD from *E. coli* was overexpressed homologously with a C-terminal hexahistidine tag and purified using affinity chromatography in 0.05% n-dodecyl-β-D-maltopyranoside (DDM, see Experimental Procedures).[8,9] SDS-PAGE analysis showed that the protein was highly pure (Figure S1A) from the single step His-column purification, indicating no further separation protocols were required. We assessed the activity of GlpD using a simple spectrophotometric assay, which uses the reduction of Q_1 as a reporter for GlpD activity (see Experimental Procedures for details). This assay showed that GlpD was active, and its activity was enhanced by the addition of a detergent-phospholipid mixture to the assay buffer (Figure S1B). Catalysis was sensitive to an established GlpD inhibitor, glyceraldehyde 3-phosphate (GA3P)[8] but importantly, not to piericidin A and antimycin A (canonical inhibitors of complexes I and III, respectively) or gramicidin A (an uncoupler used to dissipate Δp) (Figure S1C). It was also insensitive to 10 μM 2-heptyl-4-hydroxyquinoline-N-oxide (HQNO), even though it has been observed in the putative active site in a GlpD crystal structure.[8] Consistent with previous studies[8–11], the K_M values for both racemic- (*rac*)-G3P (16.7 mM) and the active stereoisomer (*sn*-G3P, 7.3 mM) were high; the maximum turnover numbers were 71 and 98 s^{-1} respectively (Figure S1D). All experiments were carried out using *rac*-G3P unless otherwise specified.

Table S1. Species comparison for complex I in the modular proteoliposome system. Representative reconstitutions provided parameters for orientation (outward facing complex I) and retention (the proportion of complex I remaining from the starting quantity). All reconstitutions were performed with 5 mg lipids, 100 μg complex I and 150 μg F_1F_O ATP synthase (3:1 molar ratio to complex I). Reconstitution parameters were obtained from the same preparations as Figure 6. Kinetic parameters were determined from the data in Figure 4 and Figure S6. Kinetic parameters for *M. musculus* were not determined. Errors given are the S.E. of the fit.

Species of complex I	Reconstitution Parameters		Kinetic Parameters			
			Forward electron transfer		Reverse electron transfer	
	Orientation	Retention	K_M (mM, membrane)	V_{max} (μmol min^{-1} (mg CI_{out})$^{-1}$)	K_M (mM, membrane)	V_{max} (μmol min^{-1} (mg CI_{out})$^{-1}$)
B. taurus	63%	60%	1.65 ± 0.20	24.25 ± 0.85	9.23 ± 1.62	0.50 ± 0.04
M. musculus	62%	72%	N.D.	N.D.	N.D.	N.D.
P. pastoris	57%	53%	0.96 ± 0.11	43.20 ± 1.21	3.35 ± 0.48	0.62 ± 0.03
Y. lipolytica	69%	64%	1.87 ± 0.13	53.73 ± 1.05	---	---

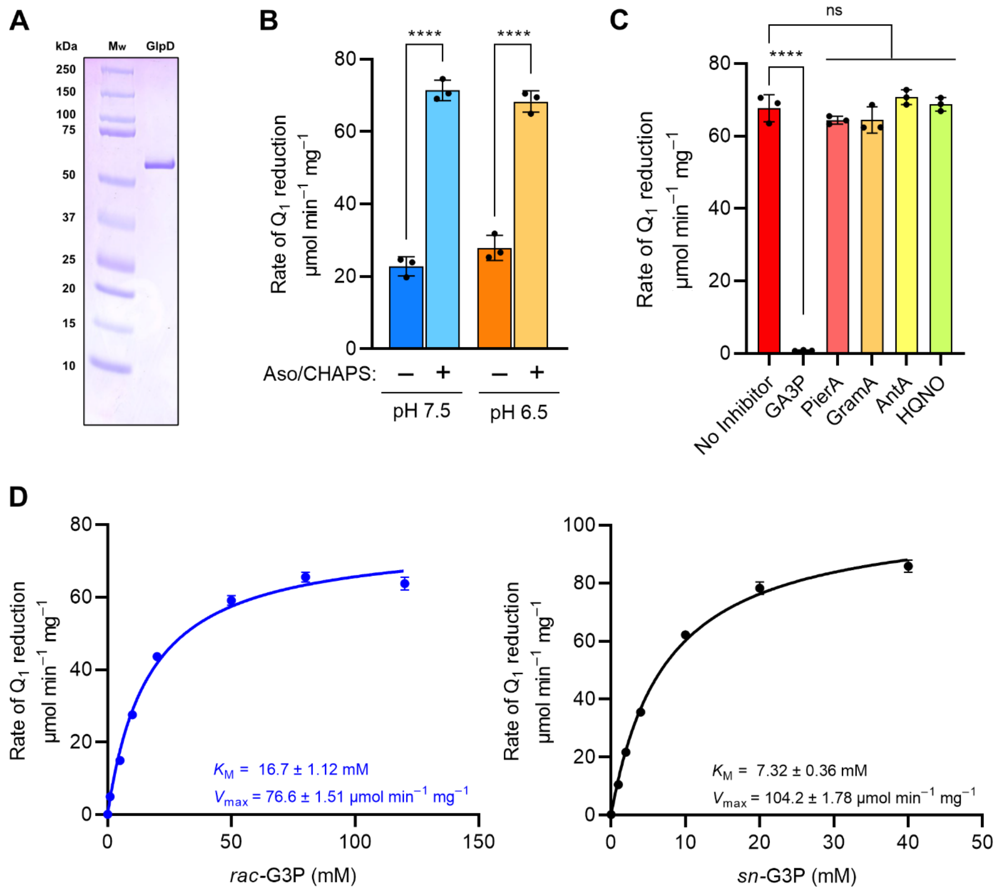

Figure S1. Characterization of GlpD overexpressed and purified from *E. coli.* (A) SDS-PAGE analysis of purified His-tagged GlpD. 1 μg isolated GlpD was loaded onto the gel and compared with a standard protein mixture (Mw; molecular weight). The single band at ~55 kDa is consistent with the expected Mw of GlpD (56,150 Da). (B) Rates of *rac*-G3P:Q_1 oxidoreduction by GlpD (0.1 μg mL^{-1}) in the standard buffers used for proteoliposome assays and in the presence and absence of 0.15% (w/v) asolectin and 0.15% (w/v) CHAPS. Turnover was initiated by addition of 120 mM rac-G3P and the reduction of 200 μM Q_1 was recorded at 275 nm (ε = 13.7 mM^{-1} cm^{-1})[12] at 32 °C. See Experimental Procedures for details. (C) The effects of candidate inhibitors on catalysis by GlpD. Assays were conducted as in panel B at pH 7.5 with asolectin/CHAPS, in the presence of glyceraldehyde 3-phosphate (GA3P, 20 mM), piericidin A (2 μM), antimycin A (2 μM), HQNO (10 μM), or gramicidin A (2 μg mL^{-1}). Note that Q-site inhibitors were used at concentrations sufficient to inhibit respiratory chain enzymes. (D) Michaelis-Menten analyses of *rac*-G3P and *sn*-G3P oxidation by GlpD (pH 7.5, 0.15% asolectin/CHAPS, 200 μM Q_1) with varying G3P concentrations. Data points are mean average values (± SD), with statistical significance for panels B and C assessed using a one-way ANOVA with Tukey's multiple comparisons correction (****$p < 0.0001$).

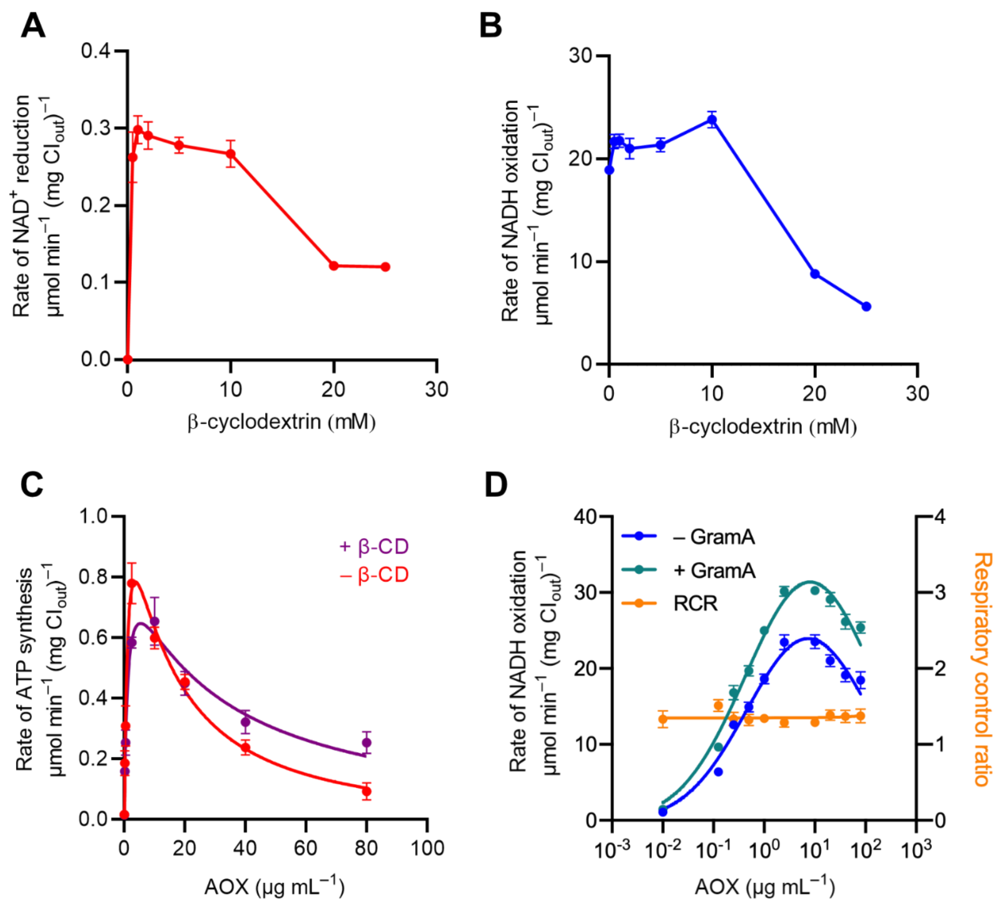

Figure S2. The effects of β-cyclodextrin on rates of catalysis by proteoliposomes. (A) Dependence of the rate of RET (GlpD-CI) on the concentration of β-CD. Rates of NAD^+ reduction were measured for proteoliposomes (5 µg CI_{out} mL^{-1}) in the presence of 1 mM $MgSO_4$, 1 mM ATP, 120 mM *rac*-G3P and 1 mM NAD^+, 100 µg mL^{-1} GlpD and varying concentrations of β-CD, following a three-minute pre-treatment with 10 µM NADH. (B) Dependence of the rate of NADH oxidation (CI-AOX) on the concentration of β-CD. Rates of NADH oxidation were measured for proteoliposomes (0.5 µg CI_{out} mL^{-1}) in the presence of 200 µM NADH, 10 µg mL^{-1} AOX and varying concentrations of β-CD. (C) Titration of AOX for NADH-coupled ATP synthesis. ATP synthesis was measured as described in Experimental Procedures in presence and absence of 2.5 mM β-CD and AOX concentrations were varied between 0.01-80 µg mL^{-1}. (D) Dependence of the rates of NADH oxidation (CI-AOX) in the presence and absence of 0.5 g mL^{-1} gramicidin A on the concentration of AOX added. Rates of NADH oxidation were measured for proteoliposomes (0.5 µg CI_{out} mL^{-1}) in the presence of 200 µM NADH, 2.5 mM β-CD and varying concentrations of AOX. The respiratory control ratio (RCR, right hand axis) was determined as the ratio of the coupled and uncoupled rates.

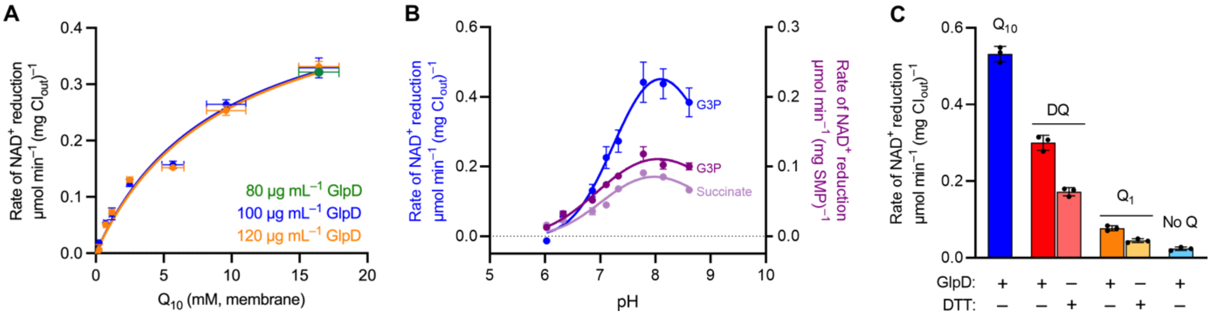

Figure S3. Characterization of proteoliposomes for reverse catalysis using Q_{10} and soluble quinones. (A) Michaelis-Menten curves of Q_{10} membrane concentration for reverse electron transfer at different concentrations of GlpD (1 mM Q_{10} (membrane) = 1 nmol Q_{10} per mg of phospholipid). The titrations were performed as described for Figure 4A. A single point using 80 µg mL^{-1} GlpD is given for the highest concentration of Q_{10}. V_{max} was determined as 0.503 ± 0.044 and 0.505 ± 0.049 µmol min^{-1} (mg CI_{out})$^{-1}$ for 100 and 120 µg mL^{-1}, respectively. K_M values were 9.13 ± 1.62 and 9.45 ± 1.85 mM for 100 and 120 µg mL^{-1}, respectively. (B) pH dependency of RET in GlpD-SMPs (purple), and proteoliposomes (blue). For CI-F_1F_O proteoliposomes, RET rates were measured under standard conditions but in the presence of 5 mM *sn*-G3P instead of 120 mM *rac*-G3P. *sn*-G3P was used to maximize the rate while minimizing the pH change from the added G3P. For succinate induced RET, rates were measured with 20 µg mL^{-1} SMPs supplemented with 80 µg mL^{-1} GlpD and treated with 1 mM $MgSO_4$, 1 mM ATP, 2.5 µM antimycin A, 2.5 mM β-CD and 5 mM succinate. For G3P induced RET, succinate was replaced by 5 mM *sn*-G3P. For all measurements, complex I was preactivated with 10 µM NADH for 3 minutes prior to RET substrate addition. All buffers contained 10 mM buffering agent, 50 mM KCl, and the buffering agents were adapted depending on the working pH (pH values at 32°C indicated in brackets): MES [6.03, 6.33], MOPS [6.86, 7.11, 7.33, 7.64] and TAPS [7.78, 8.14, 8.61]. (C) Comparison of RET by CI-F_1F_O proteoliposomes using different quinones and reductants. RET in liposomes containing Q_{10} was measured under the standard conditions described in Experimental Procedures. For measurements of soluble quinones, standard proteoliposomes were prepared in absence of Q_{10} and RET was measured using 5 µg ml^{-1} CI_{out} with 1 mM NAD^+, 1 mM ATP, 1 mM $MgSO_4$, 120 mM *rac*-G3P, 2.5 mM β-CD and 100 µM quinone (either decylubiquinone (DQ) or ubiquinone-1 (Q_1)). Quinone reduction was induced by the addition of either 80 µg mL^{-1} GlpD or 2 mM dithiothreitol (DTT).[13]

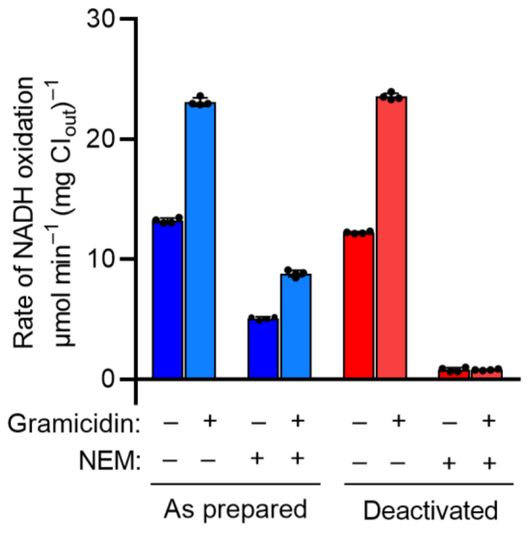

Figure S4. Measurements of the proportion of A/D complex I in CI-only proteoliposomes are unaffected by uncoupling. Complex I proteoliposomes (200 μg mL^{-1} CI_{out}) were incubated with 1 mM NEM or an equivalent volume of the DMSO vehicle (0.25%) for 20 minutes on ice before the NADH:O_2 activity was measured (see Experimental Procedures for assay details). Deactivated samples were incubated at 37 °C for 15 minutes prior to the addition of NEM or DMSO. Gramicidin A was added to the assay mixture at a concentration of 0.5 μg mL^{-1} where indicated. Comparison of the ± NEM rates for as prepared proteoliposomes gives a proportion of 38% D for both coupled and uncoupled proteoliposomes. NEM rates from deactivated proteoliposomes give a proportion of >95% D. All data are mean averages with error (± S.D.) values from 4 technical replicates.

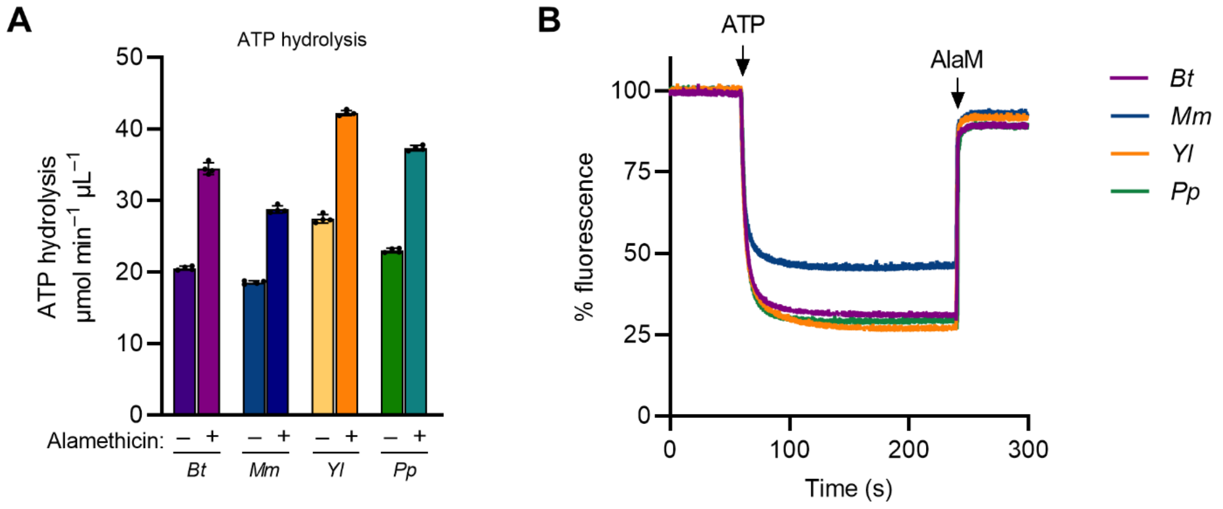

Figure S5. Characterization of ATP hydrolysis by CI-F_1F_O proteoliposomes reconstituted with complex I from different species. (A) Equivalent proteoliposomes were prepared and resuspended at the same volume. ATP hydrolysis rates were measured with 1 μL of proteoliposomes in the presence and absence of 15 μg mL^{-1} alamethicin to estimate the F_1F_O ATP synthase orientation. See Experimental Procedures for details. ATP hydrolysis rates are therefore representative of the relative retention of F_1F_O ATP synthase in these proteoliposomes. All data are mean averages with error (± S.D.) values from 4 technical replicates. (B) Formation of Δp by proteoliposomes monitored by quenching of the ACMA fluorescence during ATP hydrolysis. An equivalent volume of proteoliposomes was suspended in 2 mL assay buffer supplemented with 0.5 μM ACMA and 0.1 μM valinomycin. Proton pumping was initiated by the addition of 1 mM Mg-ATP and Δp dissipated by treatment with 15 μg mL^{-1} alamethicin (AlaM). See Experimental Procedures for further details.

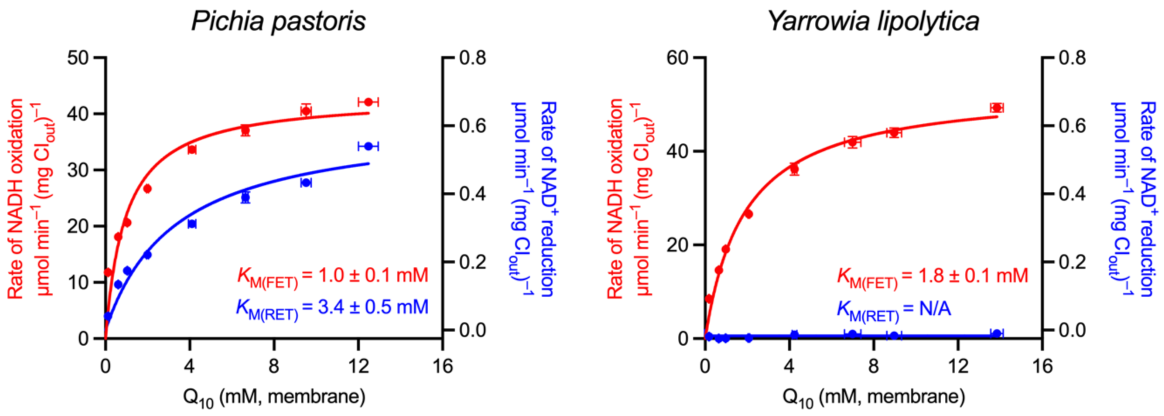

Figure S6. Q_{10} titrations for forward and reverse electron transfer in *P. pastoris* and *Y. lipolytica* complex I. Complex I and F_1F_O ATP synthase were reconstituted under standard conditions (see Experimental Procedures) with varying concentrations of Q_{10}, which were subsequently quantified. Complex I activities were measured as in Figure 4 and fit to Michaelis-Menten curves for Q_{10} concentration in the membrane (1 mM Q_{10} (membrane) = 1 nmol Q_{10} per mg of phospholipid). NADH oxidation rates represent complex I activity in the presence of the uncoupler, gramicidin A at 0.5 μg mL^{-1}. All NADH oxidation measurements are mean averages of quadruplicate technical replicates and NAD^+ reduction measurements are mean averages of triplicate technical replicates. Errors are given as ± S.E. of the fit.

Supplementary References

(1) Blaza, J. N.; Serreli, R.; Jones, A. J. Y.; Mohammed, K.; Hirst, J. Kinetic Evidence against Partitioning of the Ubiquinone Pool and the Catalytic Relevance of Respiratory-Chain Supercomplexes. *Proc. Natl. Acad. Sci. U. S. A.* **2014**, 15735–15740.

(2) Fedor, J. G.; Jones, A. J. Y.; Di Luca, A.; Kaila, V. R. I.; Hirst, J. Correlating Kinetic and Structural Data on Ubiquinone Binding and Reduction by Respiratory Complex I. *Proc. Natl. Acad. Sci. U.S.A.* **2017**, 12737–12742.

(3) Varghese, F.; Atcheson, E.; Bridges, H. R.; Hirst, J. Characterization of Clinically Identified Mutations in NDUFV1, the Flavin-Binding Subunit of Respiratory Complex I, Using a Yeast Model System. *Hum. Mol. Genet.* **2015**, 6350–6360.

(4) Kashani-Poor, N.; Kerscher, S.; Zickermann, V.; Brandt, U. Efficient Large Scale Purification of His-Tagged Proton Translocating NADH:Ubiquinone Oxidoreductase (Complex I) from the Strictly Aerobic Yeast *Yarrowia lipolytica*. *Biochim. Biophys. Acta* **2001**, 363–370.

(5) Cabrera-Orefice, A.; Yoga, E. G.; Wirth, C.; Siegmund, K.; Zwicker, K.; Guerrero-Castillo, S.; Zickermann, V.; Hunte, C.; Brandt, U. Locking Loop Movement in the Ubiquinone Pocket of Complex I Disengages the Proton Pumps. *Nat. Commun.* **2018**, 4500.

(6) Bridges, H. R.; Grgic, L.; Harbour, M. E.; Hirst, J. The Respiratory Complexes I from the Mitochondria of Two *Pichia* Species. *Biochem. J.* **2009**, 151–159.

(7) Agip, A. N. A.; Blaza, J. N.; Bridges, H. R.; Viscomi, C.; Rawson, S.; Muench, S. P.; Hirst, J. Cryo-EM Structures of Complex I from Mouse Heart Mitochondria in Two Biochemically Defined States. *Nat. Struct. Mol. Biol.* **2018**, 1–9.

(8) Yeh, J. I.; Chinte, U.; Du, S. Structure of Glycerol-3-Phosphate Dehydrogenase, an Essential Monotopic Membrane Enzyme Involved in Respiration and Metabolism. *Proc. Natl. Acad. Sci. U.S.A.* **2008**, 3280–3285.

(9) Yeh, J. I.; Du, S.; Tortajada, A.; Paulo, J.; Zhang, S. Peptergents: Peptide Detergents That Improve Stability and Functionality of a Membrane Protein, Glycerol-3-Phosphate Dehydrogenase. *Biochemistry* **2005**, 16912–16919.

(10) Walz, A.-C.; Demel, R. A.; de Kruijff, B.; Mutzel, R. Aerobic *sn*-Glycerol-3-Phosphate Dehydrogenase from *Escherichia coli* Binds to the Cytoplasmic Membrane through an Amphipathic α-Helix. *Biochem. J.* **2002**, 471–479.

(11) Orr, A. L.; Quinlan, C. L.; Perevoshchikova, I. V.; Brand, M. D. A Refined Analysis of Superoxide Production by Mitochondrial *sn*-Glycerol 3-Phosphate Dehydrogenase. *J. Biol. Chem.* **2012**, 42921–42935.

(12) Fato, R.; Estornell, E.; Di Bernardo, S.; Pallotti, F.; Castelli, G. P.; Lenaz, G. Steady-State Kinetics of the Reduction of Coenzyme Q Analogs by Complex I (NADH:Ubiquinone Oxidoreductase) in Bovine Heart Mitochondria and Submitochondrial Particles. *Biochemistry* **1996**, 2705–2716.

(13) Von Ballmoos, C.; Biner, O.; Nilsson, T.; Brzezinski, P. Mimicking Respiratory Phosphorylation Using Purified Enzymes. *Biochim. Biophys. Acta* **2016**, 321–331.
